# The Differences in the Performance Profiles Between Native and Foreign Players in the Chinese Basketball Association

**DOI:** 10.3389/fpsyg.2021.788498

**Published:** 2022-01-31

**Authors:** Xing Wang, Bin Han, Shaoliang Zhang, Liqing Zhang, Alberto Lorenzo Calvo, Miguel-Ángel Gomez

**Affiliations:** ^1^Sport Coaching College, Beijing Sport University, Beijing, China; ^2^Facultad de Ciencias de la Actividad Física y del Deporte, Universidad Politécnica de Madrid, Madrid, Spain; ^3^College of General Education, Guangdong University of Science and Technology, Dongguan, China; ^4^Division of Sport Science and Physical Education, Tsinghua University, Beijing, China

**Keywords:** performance analysis, game statistic, cluster analysis, performance profiles, Chinese Basketball Association

## Abstract

The aim of the study was to (i) use an clustering analysis method to classify and identify native and foreign basketball players into similar groups based on game-related statistics; (ii) use the Pearson’s Chi-square test to identify the key clusters that affect whether a team enters the playoffs; and (iii) use the classification tree analysis to stimulate the prediction of team ability and the construction of the team roster. The sample consisted of 422 foreign players and 1,775 native players across 9 seasons from 2011 to 2019. The clustering process allowed for the identification of nine native and six foreign player performance profiles. In addition, two clusters (*p* < 0.001, ES = 0.33; *p* < 0.001, ES = 0.28) of native players and one cluster (*p* < 0.05, ES = 0.16) of foreign players were identified that had a significant impact on team ability. These results provide alternative references for basketball staff concerning the process of evaluating native and foreign player performance in the Chinese Basketball Association.

## Introduction

The process of player selection and team formation in basketball is regarded as a key factor to achieve successful game performances (Zhang et al., 2018). The selection of players in a team is a difficult decision-making task with many dimensions (Tavana et al., 2013). Coaches and managers are required to consider their technical and tactical performances, physical and physiological characteristics, or mental and psychological factors (Arnason et al., 2004). There is a huge gap between the best and worst players in terms of technical and tactical performances in the Turkish Basketball League (Özmen, 2019). Specifically, the shooting efficiency of foreign players was greater than native players, so the selection of core players may be the key to perform successfully in the league.

Success can be mainly dependent on the combination of players with complementary skills who are capable of performing according to the demands of the playing positions (Ige and Kleiner, 1998). Previously, the majority of studies were based on traditional player positions (guards, forward, and centers) to evaluate technical and physical performances (Page et al., 2007; Sampaio et al., 2010; Pojskic et al., 2015; Gasperi et al., 2020). For example, Sampaio et al. (2006b) reported that forward were demonstrated to exhibit greater shooting efficacy inside the paint, which contributes more to game outcome than the efficacy of guards and centers. However, with the development of physical and technical performances of players, more players were able to play multiple roles on the court. Over the past few years, basketball has been considered more of a “position-less” team sport (Lutz, 2012; Samuel Kalman, 2020). Especially in the National Basketball Association (NBA), the “small ball” trend led by the Golden State Warriors promoted the revolution of modern basketball (Teramoto and Cross, 2017). The available research redefined nine playing positions of NBA players (Samuel Kalman, 2020) and predicted optimal lineups based on game-related statistics. Likewise, 13 positions were identified by the topological network in the NBA, which redefined a much finer stratification of NBA players such as “All star NBA,” “All star NBA 2nd Team,” “Paint Protectors,” and “Role Players” (Lum et al., 2013). These algorithms provided a novel perspective to evaluate game performance. Similarly, Zhang et al. (2018) reported that players from different levels of teams in the NBA were distributed in five clusters according to the anthropometric attributes and playing experience. Most players from stronger teams were allocated to the low height and weight with middle experiences group while those from weaker teams were mainly distributed in the low height and weight with low experiences group. In addition, Mateus et al. (2020) used a two-step cluster model to identify three and five different performance profiles for Euroleague and national championships, and found that better performances of players may be attributed to more playing time on court, the age or playing position, as well as the competition level. However, to our knowledge, there is no study to identify this position-less phenomenon so far in the Asian basketball leagues. Therefore, it is necessary to assist coaches in understanding the detailed characteristics of different players from Asian basketball leagues in order to improve the recruitment and selection of the core players that make a huge contribution to team success.

Based on the above considerations, the aim of the present study was to (i) use an unsupervised clustering method to classify and identify native and foreign basketball players into similar groups based on game-related statistics in the Chinese Basketball Association (CBA); (ii) identify the key player clusters that affect whether a team enters the playoffs; and (iii) use classification tree analysis to stimulate the prediction of team ability and the construction of the team roster. Our study hypothesized that different levels of teams have different team characteristics according to the refined playing positions provided by cluster analysis.

## Materials and Methods

### Data Collection and Pre-processing

The data were collected from RealGM^1^ during the season period from 2011 to 2019. A total of 3,177 individual profiles were selected, including 577 foreign players and 2,600 native players (each sample represented each player’s data in one season). Moreover, players who played less than 10 games in the whole season and had an average playing time of less than 5 min were excluded from the final sample because these players’ transformed data were regarded as unreliable statistics (Kubatko et al., 2007). Then, the datasets were finally limited to 422 foreign players and 1,775 native players. The study was conducted in accordance with the Declaration of Helsinki (WMA, 2000; Bošnjak, 2001; Tyebkhan, 2003).

### Variable Selection

The initial 39 variables were selected based on box-score and advanced statistics. The box-score statistics were transformed to per-minute statistics (original statistics/min × 40) according to players’ game duration on the court (Kubatko et al., 2007). According to the available literature, a total of 20 variables were selected for analysis (see Table 1). The top four variables [height, weight, player efficacy rating (PER), points scored per 40 min (PTS)] were excluded from clustering analysis, and were only presented as descriptive analysis (Zhang et al., 2018).

**TABLE 1:** **Selected game related variables**.

Variables (abbreviation)	Description
Height	Player height, in centimeters.
Weight	Player weight, in kilograms.
PER	Player efficiency rating statistic created by John Hollinger.
PTS	Points that a player scored per 40 min.
MIN	Minutes a player played on court per game.
2PM	The number of two-point field goals that a player has successfully made per 40 min.
2Pm	The number of two-point field goals that a player or team has unsuccessfully made per 40 min.
3PM	The number of three-point field goals that a player or team has successfully made per 40 min.
3Pm	The number of three-point field goals that a player or team has unsuccessfully made per 40 min.
FTM	The number of free throws that a player or team has successfully made per 40 min.
FTm	The number of free throws that a player or team has unsuccessfully made per 40 min.
FTRATE	The number of free throws made per field goals attempted per 40 min.
TOV	A turnover occurs when a player on offense loses the ball to the defense per 40 min.
AST	An assist occurs when a player completes a pass to a teammate that directly leads to a field goal per 40 min.
STL	A steal occurs when a defensive player takes the ball from a player on offense per 40 min.
BLK	A block occurs when an offensive player attempts a shot, and a defensive player tips the ball, blocking their chance to score per 40 min.
PF	The total number of fouls that a player has committed per 40 min.
OREB	The number of rebounds that a player has collected while they were on offense per 40 min.
DREB	The number of rebounds that a player has collected while they were on defense per 40 min.
USG	The percentage of plays utilized by a player while he is in the game.

In order to test the validity of datasets, a sub-sample of 50 games (at least five games in each season) was randomly selected and observed by two experienced analysts (basketball video coordinators with more than 5 years of experience in basketball performance analysis) by using Catapult Vision. The results were contrasted with the gathered data in the website in order to provide internal validity (ICC = 0.91) and external validity using generalizability analysis (generalizability coefficient, e2 = 0.96; and reliability coefficient, Φ = 0.65) (Blanco Villaseñor et al., 2014; Hernández-Mendo et al., 2016; Royuela et al., 2017; Reigal et al., 2020). There was formal approval of all procedures from the Local Institution of Research Review Board.

### Statistical Analysis

Firstly, model-based cluster analysis within Gaussian finite mixture models (GMM) was carried out to classify native and foreign players into different groups according to selected variables (Lutz, 2012; Samuel Kalman, 2020). GMM clustering results in a soft assignment, indicating the probability that each player belongs to a cluster (Fraley and Raftery, 1998). The algorithm of GMM clustering calculates the maximum-likelihood estimate (MLE) of Equation 1 to find the optimal distribution underlying the unlabeled data. The above procedure used the “mclust” package in R (Scrucca et al., 2016).

Secondly, we used obtained player clusters to build a lineup of each team. Since the CBA official bans trading native players during the season, the lineup of native players consisted of all native players belonging to the team, and we counted the number of each cluster (including starters and non-starters). As to foreign players, since teams had a limit on the number of foreign players they could replace during the season and only two or three foreign players were allowed at the same time, the lineup of foreign players consisted of foreign players whose number of games played was in the top 2 in the whole season. The team lineup was combined using native and foreign lineups as follows:


L⁢i⁢n⁢e⁢u⁢p=N⁢1+N⁢2+N⁢3+N⁢4+N⁢5+N⁢6+N⁢7+N⁢8+N⁢9+F⁢1+F⁢2+F⁢3+F⁢4+F⁢5

Where each cluster variable represented the number of players belonging to this cluster in the team.

According to the team rankings of each season, the teams were classified into “playoffs teams” and “non-playoffs teams.” Then, a descriptive and inferential analysis was performed using the crosstabs command. The Pearson’s Chi-square test was used to analyze the effects between team abilities and the number of each player clusters in the team lineup. Each player cluster in each team was considered an independent sampling unit, the interaction with teammates was disregarded. Effect sizes (ES) were calculated using the Cramer’s *V*-test and their interpretation was based on the following criteria: 0.10 = small effect, 0.30 = medium effect, and 0.50 = large effect (Volker, 2006). The above procedure was run using the IBM SPSS statistical software for Windows, version 20.0 (Armonk, NY: IBM. Corp.).

Thirdly, a classification tree analysis (CART) was used to simulate the decision-making process of team lineup construction. The CART technique splits the sample into segments that are as homogeneous as possible in relation to the dependent variable (playoffs/non-playoffs). Since the algorithm is non-parametric and non-linear, it is often able to uncover complex interactions between predictors which may be difficult or impossible to uncover using traditional multivariate techniques (Lewis, 2000). This statistical analysis was performed using the “Rpart” package in R (Computing, 1991; Therneau et al., 2015), version 4.0.2.

## Results

The model-based clustering analysis allowed us to obtain nine clusters of native players (N1-N9) and six clusters of foreign players (F1–F6).

### Defining the Nine Playing Positions of Native Players

**FIGURE 1:**
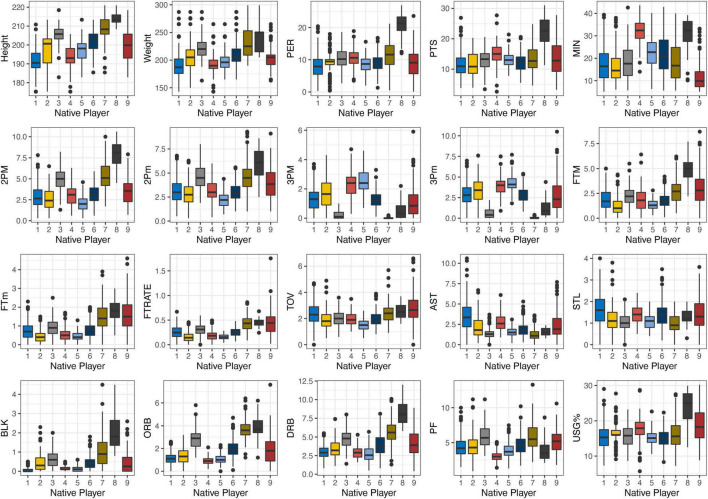
Descriptive statistics of different performance profile clusters in native players.

Figure 1 presents the native players’ performance profiles, and the definitions of the nine native players’ clusters are as follows:

N1——“Floor General”: the average height and weight of N1 were the lowest among all clusters but with the highest in assists and steal. Most of the point guards who prefer pass-first were grouped, most of this cluster were playmakers.
N2——“Sixth Man” had the second lowest average playing time (15 min per game) among all clusters but with high usage.
N3——“Rotation Big” was one of the tree clusters with average height over 205 cm but the average playing time was the lowest among the clusters.
N4——“Shooting Guard”: the average playing time of N4 was the second highest among all nine clusters, with high average 3-pointers made and missed but low PER.
N5——“Three-Point Shooting Forward” had the same average 3-pointers made and miss statistics but with the lowest 2-pointers made and missed among all clusters.
N6——“Skilled Forward” was slightly higher than average in all game-related statistics but with no outstanding feature.
N7——“Defensive Big” was slight higher than N3, with higher offensive rebound and defensive rebound.
N8——“Dominant Center” was the highest in most statistics (i.e., height, PER, PTS, 2-pointers made, USG%) but low in assists, steal, and 3-pointers made and missed.
N9——“Bench Marginal Players”: players from the bench always played below 10 min in garbage time. Most of the clusters were young players.

### Defining the Six Playing Positions of Foreign Players

**FIGURE 2:**
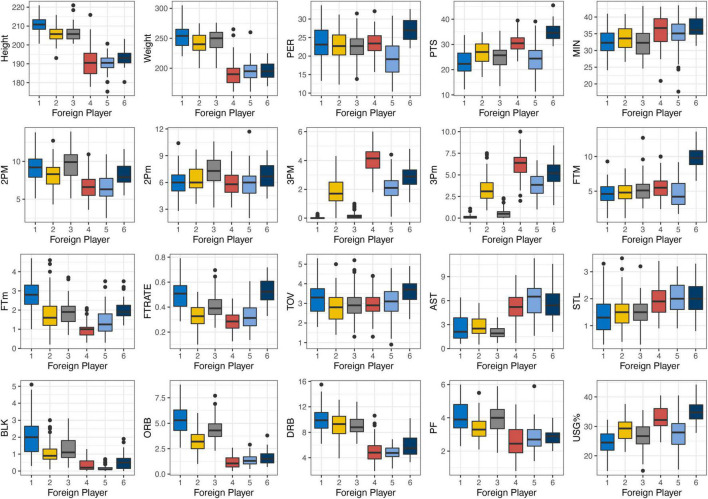
Descriptive statistics of different performance profile clusters in foreign players.

Figure 2 presents the foreign players’ performance profiles, and the definitions of six clusters in foreign players are as follows:

F1——“Traditional Centers”: players whose average height and weight were the highest among all clusters and excellent in defensive rebound, offensive rebound, and blocking shots.
F2——“Space Stretch Forward”: the average height of F2 was more than 200 cm and had good 3-pointer shoot ability, meanwhile they could guarantee some defensive rebounds. These players stood outside the three-point line on offense most of the time, which meant they did not have many opportunities to take offense rebounds than other big players.
F3——“Mid-Range Skilled Forward”: players whose role was to get the ball at midrange and low post areas according to its two-point field goals variables were able to create offensive opportunities by isolation and jump shot skill with few assists.
F4——“Three-Point Shooting Guards”: the small players who had a high-level 3-pointer shooting ability and infinite shooting privilege was evident on three-point field goals made and missed variables but had the lowest free throws rate. In addition, these players had the second highest usage rate among all foreign player clusters.
F5——“Traditional Point Guard”: this cluster includes players with the highest assists and steals but were average at other variables especially in terms of shooting, representing the Traditional Point Guard who prefers to be a team leader by assisting teammates to score than scoring by themselves. It makes them less outstanding on PER compared to other cluster players.
F6——“Dominant Point Guard” includes small players who operate with the ball in their hands and play more aggressively than the Traditional Point Guard. It is worth mentioning that these players are good at scoring by drawing fouls which ensures that they accumulate more free throw field goals than others.

### Crosstabs Analysis in Team Composition

The sample distribution of the number of native player clusters for a team is presented in Table 2. It shows that in native players only N7 Defensive Big was statistically significant (*P* < 0.001). When a team had more than two Defensive Big players it was easier to reach playoffs (30.6% compared to 25.3% when there were two Defensive Big players in the team; 18.1% compared to 2.2% for two; 1.4% compared to 0.0% for four). Conversely, the team had a low probability of entering the playoffs when no or only one Defensive Big player was in the team lineup (12.5% compared to 28.6% for 0; 37.5% compared to 44.0% for 1).

**TABLE 2:** Frequency distribution (%) of team ability according to the number of native player clusters (crosstab command: Pearson’s Chi-square, degrees of freedom, significance, expected frequency distribution, and effect size).

	Playoffs *n* = 72		Non-playoffs *n* = 91						
Number of players	%	*n*	%	*n*	χ^2^	df	*P*	EFD	ES
**N1 Floor general**									
0	5.6	4	1.1	1	8.099	6	0.261	1.32^†^	0.22
1	27.8	20	19.8	18					
2	26.4	19	22.0	20					
3	19.4	14	25.3	23					
4	16.7	12	24.2	22					
5	4.2	3	4.4	4					
6	0.0	0	3.3	3					
**N2 Sixth man**									
0	62.5	45	52.7	48	8.611	5	0.127	1.32^†^	0.22
1	23.6	17	30.8	28					
2	5.6	4	9.9	9					
3	1.4	1	5.5	5					
4	2.8	2	1.1	1					
5	4.2	3	0.0	0					
**N3 Rotation big**									
0	24.7	25	25.3	23	2.183	3	0.57	3.09^†^	0.11
1	41.7	30	42.6	42					
2	20.8	15	23.1	21					
3	2.8	2	5.5	5					
**N4 Shooting guard**									
0	33.3	24	37.4	34	0.307	3	0.933	0.88^†^	0.043
1	55.6	40	52.7	48					
2	9.7	7	8.8	8					
3	1.4	1	1.1	1					
**N5 Traditional point guard**									
0	18.1	13	18.7	17	5.009	3	0.171	7.06	0.17
1	43.1	31	39.6	36					
2	34.7	25	27.5	25					
3	4.2	3	14.3	13					
**N6 Skilled forward**									
0	12.5	9	8.8	8	8.535	5	0.109	1.32^†^	0.22
1	31.9	23	29.7	27					
2	41.7	30	33.0	30					
3	9.7	7	17.6	16					
4	1.4	1	9.9	9					
5	2.8	2	1.1	1					
**n7 Defensive big**									
0	12.5	9	28.6	26	17.896	4	0.001**	0.44^†^	0.33
1	37.5	27	44.0	40					
2	30.6	22	25.3	23					
3	18.1	13	2.2	2					
4	1.4	1	0.0	0					
**N8 Dominant center**									
0	75.0	54	93.4	85	13.226	2	0.001**	0.44^†^	0.28
1	25.0	18	5.5	5					
2	0.0	0	1.0	1					
**N9 Bench marginal player**									
0	55.6	40	59.3	54	2.006	4	0.799	0.44^†^	0.11
1	31.9	23	30.8	28					
2	11.1	8	6.6	6					
3	1.4	1	2.2	2					
4	0.0	0	1.1	1					

*P < 0.05; **P < 0.01; EFD, expected frequency distribution; ^†^When EFD was below 5 or the variable includes values below 1%, the Fisher’s exact test was applied; ES, effect size.

In addition, N8 Dominant Center had the same positive role as N7 Defensive Big. The result showed that when a team had one Dominant Center player the team had more chances to make playoffs (25.0% compared to 5.5% for one Dominant Center player in the team). But when there was a lack of Dominant Center players in the team, it was more difficult to make the playoffs (75.0% compared to 93.4%).

The result for foreign player clusters (Table 3) showed that only F6 Dominant Point Guard was significantly related to team ability. When there was a Dominant Point Guard foreign player in the team, the probability of the team entering the playoffs was lower than not making the playoffs (27.5% compared to 13.9%). Conversely, the team had a high probability of entering the playoffs when no Dominant Point Guard was in the team lineup (86.1% compared to 72.9%).

**TABLE 3:** Frequency distribution (%) of team ability according to the number of foreign player clusters (crosstab command: Pearson’s Chi-square, degrees of freedom, significance, expected frequency distribution, and effect size).

	Playoffs *n* = 72		Non-playoffs *n* = 91						
Number of players	%	*n*	%	*n*	χ^2^	df	*P*	EFD	ES
**F1 Traditional center**									
0	81.9	59	75.8	69	0.893	1	0.345	15.46	0.07
1	18.1	13	24.2	22					
**F2 Space stretch forward**									
0	59.7	43	62.6	57	1.919	2	0.513	0.88^†^	0.11
1	40.3	29	35.2	32					
2	0.0	0	2.2	2					
**F3 Mid-range skilled forward**									
0	56.9	41	56.0	51	0.152	2	1	3.53^†^	0.03
1	38.9	28	38.5	35					
2	4.2	3	5.5	5					
**F4 Three-point shooting guard**									
0	50.0	36	57.1	52	3.745	2	0.198	1.33^†^	0.15
1	50.0	36	39.6	36					
2	0.0	0	3.3	3					
**F5 Traditional point guard**									
0	58.3	42	72.5	66	4.201	2	0.0938	1.33^†^	0.16
1	40.3	29	25.3	23					
2	1.4	1	2.2	2					
**F6 Dominant point guard**									
0	86.1	62	72.5	66	4.399	1	0.036*	15.46	0.16
1	13.9	10	27.5	25					

*P < 0.05; **P < 0.01; EFD, expected frequency distribution; ^†^When EFD was below 5 or the variable includes values below 1%, the Fisher’s exact test was applied; ES, effect size.

**FIGURE 3:**
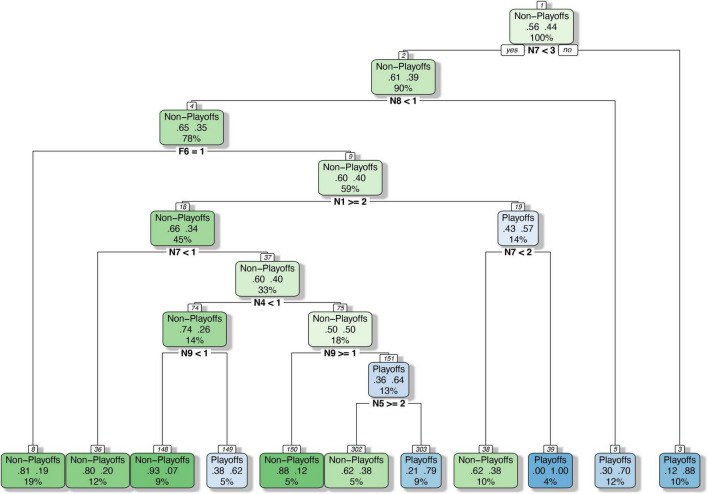
Classification and regression tree analysis of team abilities.

The classification and regression tree analysis included both native and foreign player cluster variables in the statistical model. Figure 3 shows that, after pruning by the minimum error algorithm, a total of 21 nodes were defined which included 10 parent nodes and 11 leaf nodes. Each parent node was split by a player cluster variable. The splitting variables for the top 3 parent nodes were the same as the significant variables provided by crosstab analysis (N7 Defensive Big, N8 Dominant Center, F6 Dominant Point Guard). In addition, another four variables (N1 Floor General, N4 Shooting Guard, N9 Bench Marginal Player, N5 Three-Point Shooting Forward) were also considered as splitting variables in the final tree. Each leaf node provided the probability of the team in this cluster of entering the playoffs and the probabilities of six nodes (8, 36, 148, 150, 302, and 38) were lower than 50%, and five nodes (149, 303, 39, 5, and 3) were more than 50%. Among them, the lowest probability of entering the playoffs was node 148 in which only 7% of teams in this node were likely to enter the playoffs. In contrast, 100% of the teams in node 39 could enter the playoffs, but the sample size was only 4% of the total sample.

The root node (node 1) was split by N7 Defensive Big. High probabilities (88%) to make the playoffs were evident when the obtained values for N7 Defensive Big were higher than 2.5 (node 3) and, conversely, lower chances (39%) to make the playoffs were seen when the obtained values for assists were equal or lower than 2 (node 2). Based on the number of N8 Dominant Centers, node 2 was split into node 4 and leaf node 5. Leaf node 5 showed that in the team that had no more than two N7 Defensive Bigs, if there were more than one N8 Dominant Center, the probability of this team making the playoffs would be 70%. Node 4 was further split into leaf node 8 and node 9 by the number of F6 Dominant Point Guards. When there were less than two N7 Defensive Bigs and no N8 Dominant Centers in the team, and if the team had an F6 Dominant Point Guard in the lineup, the team had an 81% probability of not making the playoffs.

## Discussion

The aim of the present study was to (i) use an unsupervised clustering method to classify and identify native and foreign basketball players into similar groups based on game-related statistics; (ii) identify the key clusters that affect whether a team enters the playoffs; and (iii) use classification tree analysis to stimulate the prediction of team ability and the construction of the team roster. It was expected that some players would have a significant impact on the strength of the team (i.e., all-star players, scoring players, or defensive players). Our results revealed a discrepancy of individual performance with nine clusters identified for the native players and six clusters for the foreign players. Furthermore, three clusters of players highlighted significantly different distributions in playoffs and non-playoffs teams. These findings will be of extreme importance for coaches and managers in CBA when recruiting players and building team lineups based on players’ strengths and weaknesses, playing position, and nationality.

### Difference Between Player Clusters in the Roster

Based on lineups built by new players’ clusters, the crosstabs command analysis identified that the number of players from three clusters showed significant differences between playoffs and non-playoffs teams. In native players, the most important playing position was Dominant Center which is linked with previous studies reporting that the most prominent performance characteristics of Dominant Centers are closely related to the team’s wins and losses (i.e., two-point field goals made, free throws made, defensive rebounds, and blocked shots) in high-level competition (Çene, 2018). However, talented players can be defined as Dominant Centers and are extremely rare in the league (1.75% of all native players) which means that most teams usually cannot have this cluster of players. Thus, a team that does not have Dominant Centers can only use Defensive Big players as substitutes. The number of this cluster is also significantly different between playoffs and non-playoffs teams. Though Defensive Big is lower than Dominant Center in some offensive variables (i.e., PTS, PER, two-point field goals, free throws made, and USG%), these players have a similar effect to Dominant Center on defensive variables (blocks and defensive rebounds). This finding confirms the conclusions of previous research that centers from winning teams secure more defensive rebounds and make more blocks in contrast to players from the same position in losing teams (Zhang et al., 2019). For foreign players, our study found that Dominant Point Guards are more distributed in non-playoffs teams than playoffs teams. In terms of personal game performances, these players contribute the most to the teams’ wins with higher PTS and USG% than other clusters (Hollinger, 2005; Sampaio et al., 2006a). However, basketball is a competitive team sport that emphasizes teamwork (Melnick, 2001) and according to Oliver’s offensive skill curves (Oliver, 2004), the more possessions a team has, the less offense efficiency it has, and then a critical performance occurs. In addition, the high USG% also reflects the imbalance in the overall strength of the team. When teammates cannot score on the court, a player like a Dominant Point Guard has to take over more offensive possessions to win.

### Classification and Regression Tree Analysis

Our study used the classification and regression tree model to simulate the prediction of team ability and the construction of the team roster. The results identified that the first two clusters that had the greatest impact on team ability were in native players (N7 Defensive Big and N8 Dominant Centers), and a total of six clusters in native players were selected in the tree but only one cluster in foreign players. This finding is similar to the conclusion that Ozmen got in his research (Ozmen, 2012), that efficiency of foreign players in top teams is no different than that of foreign players in regular (non-top) teams, whereas, native players in top teams are more efficient than native players in other teams. According to the previous clustering result, the main game-related characteristics of Defensive Big and Dominant Center were offensive rebound and defensive rebound, which means if a team could have more such players in their rotation, they can guarantee rebounds at any time during the game. In fact, the defensive rebound is the most important variable for the game outcome (Lorenzo et al., 2010; Gómez et al., 2017). For N1 Floor General, the average PER of this cluster of native players was the lowest. If there are too many Floor Generals in the team roster, inevitably it will pull down the efficiency of the team (Ozmen, 2012). The same explanation can also be used on N4 whose average PER was third among all native player clusters, preceded only by Dominant Center and Defensive Big. However, for the foreign player cluster with particularly high numbers of F6 Dominant Point Guards, the CART tree recommended that the playoff teams try not to recruit these players.

There are limitations in the current research that should be considered in further studies concerning players’ and team’s performance profiles. Firstly, due to a lack of shooting type and area variables, the new positions obtained by the clustering method cannot fully reflect the ability and style of each player. Secondly, psychological variables and situational variables are also important factors that affect the decision-making of coaches and managers. Finally, after clustering new player positions, the team’s lineup just represented the roster of the team in the whole season but not all the players could play in every game. Thus, future studies can be developed based on the data of each game or 5-man lineup on the court and delve into the coach’s on-the-spot substitution decision-making.

## Conclusion

In summary, this study provides a new understanding of playing positions (Floor General, Sixth Man, Rotation Big, Shooting Guard, Three-Point Shooting Forward, Skilled Forward, Defensive Big, Dominant Center, and Bench Marginal Player in native players; Traditional center, Space Stretch Forward, Mid-Range Skilled Forward, Three-Point Shooting Guard, Traditional Point Guard, and Dominant Point Guard in foreign players) and team lineup composition in the CBA. Having a high-level of native big players is the key factor for a team entering the playoffs while the most negative impact is a Dominant Point Guard foreign player. Therefore, basketball coaches and managers will benefit from being aware of these results, particularly to set up teams and optimize preparation for individual player clusters in order to improve game performances of the players and teams.

## Data Availability Statement

Publicly available datasets were analyzed in this study. This data can be found here: https://basketball.realgm.com/international/league/40/Chinese-CBA/stats.

## Author Contributions

XW, BH, and SZ contributed to conception of the study. XW organized the database. BH performed the statistical analysis. LZ wrote the first draft of the manuscript. M-ÁG and ALC supervised the design and reviewed the manuscript. All authors contributed to manuscript revision, read, and approved the submitted version.

## Conflict of Interest

The authors declare that the research was conducted in the absence of any commercial or financial relationships that could be construed as a potential conflict of interest.

## Publisher’s Note

All claims expressed in this article are solely those of the authors and do not necessarily represent those of their affiliated organizations, or those of the publisher, the editors and the reviewers. Any product that may be evaluated in this article, or claim that may be made by its manufacturer, is not guaranteed or endorsed by the publisher.

## Footnotes

1
http://basketball.realgm.com
